# Modelling the impact of social protection on tuberculosis: the S-PROTECT project

**DOI:** 10.1186/s12889-018-5539-x

**Published:** 2018-06-26

**Authors:** D. Boccia, W. Rudgard, S. Shrestha, K. Lönnroth, P. Eckhoff, J. Golub, M. Sanchez, E. Maciel, D. Rasella, P. Shete, D. Pedrazzoli, R. Houben, S. Chang, D. Dowdy

**Affiliations:** 1 Faculty of Epidemiology and Population Health, London School of Hygiene and Tropical Medicine, London, UK; 2 Department of Epidemiology, Johns Hopkins Bloomberg School of Public Health, Baltimore, USA; 3 Department of Public Health Sciences, Karolinska Institutet, Stockholm, Sweden; 4 Institute for Disease Modeling, Bellevue, USA; 5 Department of Medicine, Epidemiology & International Health, Johns Hopkins Bloomberg School of Public Health, Baltimore, USA; 6 Federal University of Brasilia, Brasilia, Brazil; 7 Federal University of Espírito Santo, Maruipe, Vitória, Brazil; 8 Oswaldo Cruz Foundation (FIOCRUZ), Brasília, DF Brazil; 9 Global Tuberculosis Programme, World Health Organization, Geneva, Switzerland; 10 Division of Pulmonary and Critical Care Medicine, University of California San Francisco, San Francisco, USA; 11 TB Modelling Group, TB Centre and CMMID, London School of Hygiene and Tropical Medicine, Faculty of Epidemiology and Population Health, London, UK

**Keywords:** Evaluation, Cash transfers, Tuberculosis, Control, Brazil

## Abstract

**Background:**

Tackling the social determinants of Tuberculosis (TB) through social protection is a key element of the post-2015 End TB Strategy. However, evidence informing policies are still scarce. Mathematical modelling has the potential to contribute to fill this knowledge gap, but existing models are inadequate. The S-PROTECT consortium aimed to develop an innovative mathematical modelling approach to better understand the role of social protection to improve TB care, prevention and control.

**Methods:**

S-PROTECT used a three-steps approach: 1) the development of a conceptual framework; 2) the extraction from this framework of three high-priority mechanistic pathways amenable for modelling; 3) the development of a revised version of a standard TB transmission model able to capture the structure of these pathways. As a test case we used the Bolsa Familia Programme (BFP), the Brazilian conditional cash transfer scheme.

**Results:**

Assessing one of these pathways, we estimated that BFP can reduce TB prevalence by 4% by improving households income and thus their nutritional status. When looking at the direct impact via malnutrition (not income mediated) the impact was 33%. This variation was due to limited data availability, uncertainties on data transformation and the pathway approach taken. These results are preliminary and only aim to serve as illustrative example of the methodological challenges encountered in this first modelling attempt, nonetheless they suggest the potential added value of integrating TB standard of care with social protection strategies.

**Conclusions:**

Results are to be confirmed with further analysis. However, by developing a generalizable modelling framework, S-PROTECT proved that the modelling of social protection is complex, but doable and allowed to draw the research road map for the future in this field.

**Electronic supplementary material:**

The online version of this article (10.1186/s12889-018-5539-x) contains supplementary material, which is available to authorized users.

## Background

The End TB Strategy has placed TB within the new sustainable development agenda [1] under which health and development are considered to be profoundly interlinked. No sustainable health or development goal can be achieved without a truly multisectoral approach. The alignment between the End TB Strategy and the SDGs is reflected both in this underlying philosophy, as well as the proposed targets and strategies. Social protection, which sits at the intersection between health and social interventions, can contribute to both poverty elimination and important health goals, include the goal to End TB [2].

Social protection has been defined as a wide range of policies to move people out of extreme poverty and achieve sustainable inclusion and economic growth [3]. Among them, cash transfers interventions (CTIs), whether conditional or unconditional, have become some of the most popular forms of social protection approaches [4] and they are also the main focus of this paper.

CTIs are increasingly regarded as a potentially powerful tool to enhance TB prevention (by allowing better resilience to TB infection and disease that result from improved material living conditions); TB care (by enabling better and more timely access to quality TB care); and TB support (by mitigating the TB-related catastrophic costs) [5]. Evidence to support this postulated impact are slowly accumulating [6–12]; however the existing knowledge base is still insufficient to derive conclusions about the quantitative impact of CTIs on TB and the mechanisms through which this impact is likely to happen. While randomised trials would be the gold standard for answering these questions, they can be complex, expensive, lengthy, and potentially unethical. Mathematical models could play an important role in filling these knowledge gaps. Unfortunately few modelling exercises have explored the degree to which socioeconomic interventions affect TB risks, and to which social protection interventions can reduce TB burden by modifying these determinants [13, 14]. This situation largely reflects the challenges of assembling the interdisciplinary teams necessary to construct such models and of describing the complex web of causal factors through which CTIs might impact TB burden and distribution using quantitative terms [15]. Although several conceptual frameworks have been developed to describe these mechanisms [16], these frameworks have rarely been tested with epidemiological studies to construct mathematical models. Furthermore, the quality and accessibility of epidemiological data linking TB epidemiologic data and social protection in general is often sparse. Thus, while the potential effect of CTIs on TB control is deemed potentially large, quantifying that effect in the context of a tractable model may be challenging.

The S-PROTECT (Social PROTection to Enhance the Control of Tuberculosis) consortium was created to respond to these challenges: we developed a combined conceptual-quantitative modelling framework through which existing empirical data could be used to inform estimates of the effect of CTIs on TB control. Importantly, in this paper we do not attempt to reach conclusions on the impact of CTIs on TB; rather, we illustrate methodological steps during the first 12 months of the project and highlight the strengths and limitations of the approach taken by presenting some preliminary mathematical modelling findings. We conclude by discussing the main challenges encountered and how they can inform the future research agenda on social protection modelling.

## Methods

### The S-PROTECT conceptual framework

The S-PROTECT Modelling Consortium aimed to bring together an interdisciplinary team of experts (including mathematical modellers, social epidemiologists, decision-makers, and local implementers) to develop a mathematical model able to capture the various processes through which CTIs may influence TB epidemiology and control. Critical steps in the development of this tool included: a) advancing a theory-based conceptual framework describing the mechanism through which social protection might affect TB outcomes and then translating this conceptual framework into a simplified version suitable for modelling purposes; b) using a modified Delphi process to rank mechanistic pathways within this conceptual framework that would be most suitable for incorporation into a mathematical model. c) developing a mathematical model best reflecting the structure of these pathways. We used as a test case *Bolsa Familia Programme (BFP)*, the Brazilian national conditional cash transfer programme (Table 1).

**Table 1:** The Bolsa Familia Programme (BFP) [30]

• BFP aims to enhance the human capital of poor Brazilian citizens through an increased utilisation of public services and break the inter-generational transmission of poverty. • With its 46.5 million beneficiaries around the country, BFP is today the largest conditional cash transfer program in the world. • BFP targets extremely poor households earning between US $25–50 per person per month. • Depending on the household composition, the monthly cash benefits range from US $12 to a maximum of few hundreds US dollars depending on household composition. • Transfers are given under three conditions: 1) attendance of prenatal and postnatal monitoring; 2) access to nutrition and vaccination monitoring for their children aged 0–7 years; 3) school attendance.	

#### The conceptual underpinning

**Fig. 1:**
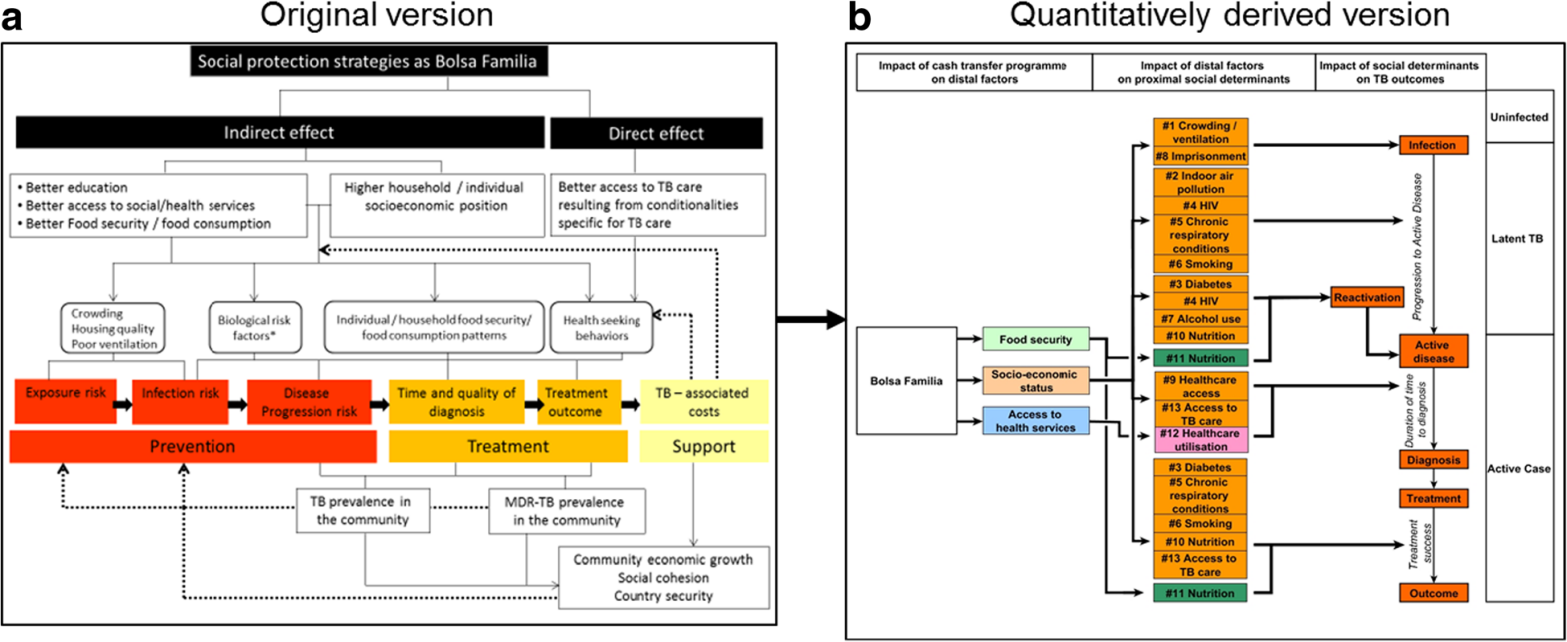
Conceptual framework: the original version and the quantitative translation into 13 separate pathways. In its original version (Fig. 1**a**) this framework assumes that social protection interventions such as the Bolsa Familia Programme can improve TB outcomes either indirectly by influencing the distal/structural social determinants of TB (e.g., living conditions, food security), or directly by making support conditional on health-seeking behaviours relevant for TB (e.g. TB testing and preventive therapy among household contacts, TB treatment completion, BCG vaccination in children). Indirect effects on distal social determinants can alter a number of proximal mediators (e.g., indoor air pollution, malnutrition, co-morbidities) known to be associated with TB infection, progression, disease severity, or TB outcomes (including economic impact, albeit this was not accounted for in this initial modelling effort). To the extent that these associations are causal, and depending of the population targeted, reducing the prevalence of these mediators should improve TB outcomes accordingly. Fig 1**b** represents graphically the quantitatively derived version of this framework outlining the 13 pathways that were prioritised and extracted specifically for Bolsa Familia in Brazil. As explained in the text, three levels of impact can be identified (outlined at the top of figure: 1) the impact of social protection on distal factors (e.g. household socioeconomic position); 2) the impact of distal factors on more proximal social determinants of TB (i.e. listed from 1 to 13 depending on the specific pathway); and 3) the impact of these factors on TB outcomes (e.g. infection or disease). When social protection schemes are developed to have direct effects on TB outcomes themselves (e.g. in TB-specific interventions),(HYPERLINK "" \l "_ENREF_17" \o "Boccia, 2016 #1990" 17) the first two levels (1 and 2) can be bypassed; however, in the much more common situation where social protection is designed at programmatic level (like BFP) to improve the distal structural determinants of health, all three levels must be considered

The underlying conceptual framework used by S-PROTECT was adapted from Boccia et al. [17] This framework (Fig. 1) attempts to map the pathway through which social protection may have an influence on the various stages of TB natural history.

Within this framework three distinct levels of impact can be distinguished:

*Level 1* - Impact of social protection on distal factors/structural determinants of health [18].

*Level 2* - Impact of distal factors on proximal mediators/social determinants of TB [18].

*Level 3* - Impact of proximal mediators on TB outcomes.

#### Prioritizing the mechanistic pathways

In order to incorporate the above levels of effect into a quantitative model, we used the tool of *mechanistic pathways.* Specifically, through a consensus review process, the S-PROTECT team first brainstormed and then prioritised a set of pathways that were consistent with the original conceptual framework but that could be quantified at each of the three levels of effect. Figure 1b and Table 2 show 13 pathways - an illustrative rather than comprehensive list. Each mechanistic pathway can be conceptualised as a quantitative adaptation of a component of the framework in Fig. 1; if the entirety of these pathways and their overlap could be identified and quantified, then the full effect of social protection on TB outcomes (except for financial outcomes) could be estimated. Since this is not possible, we decided instead to prioritise individual pathways for further investigation, understanding that we are thereby excluding the effects of non-prioritised pathways, as well as any synergies (or antagonism) between individual pathways when those pathways are realised together.

**Table 2:**
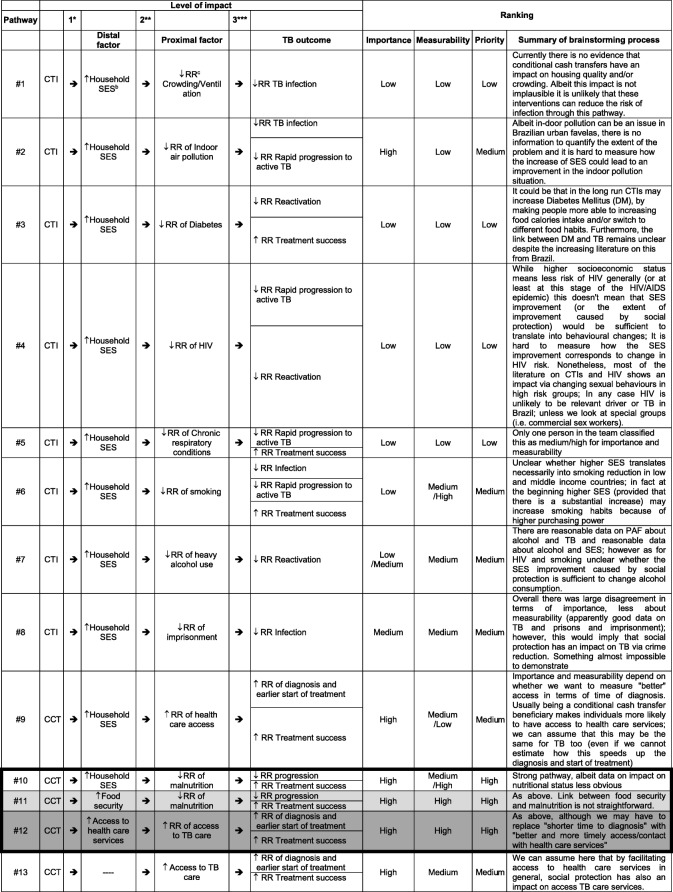
Pathways extracted from the conceptual framework and pathways ranking^a^

*Impact of social protection on distal factors

**Impact of distal factors on social determinants of TB

***Impact of social determinants of TB on TB outcomes

^a^Pathway #10, #11 and #12 were identified as high priority pathways: These pathways start with three different distal factor, including household socioeconomic position, food security and access to health care services respectively for pathway #10, #11 and #12. According to pathway #10 and #11, household socioeconomic position and food security can influence the risk of household members malnutrition which in turns affects their likelihood of TB progression (including reactivation) and TB treatment success. As for pathway #12 better access to health care services means also better access to TB care services and thus better and more timely TB diagnosis and care and ultimately better TB treatment outcome

^b^Household SES = Household Socioeconomic Status

^c^RR = Relative risk

In prioritizing among the 13 pathways for further exploration with the modelling framework, we used two criteria: (1) *importance* from the perspective of the likely effect of the pathway on the epidemiology of TB (and specifically in Brazil); and (2) *measurability* in terms of whether data (whether from the published literature or from publicly available sources) might currently exist to inform quantitative estimates of association at each level of the pathway. Pathways were ranked as either low, medium, or high priority. Differences between team members were resolved by consensus, resulting in the ranking displayed in Table 2.

Through this process three priority pathways were identified: pathways #10, #11 and #12 (Table 2). These three high priority pathways were populated with evidence obtained from three sources of data: a) published effect estimates from the literature; b) de novo analysis of publicly available data; and when no other estimate could be identified, c) assumptions made by experts in the field of social protection and TB. The data collection and synthesis approach as well as data assumptions are described respectively in the online Additional file 1: Appendix 1 and 2.

Pathway #10, was then further developed for inclusion into the quantitative modelling framework (Table 3 and Table 4).

**Table 3:** Measures of impact for pathway #10

Level 1: Impact of CCT like BFP on Household Socioeconomic Status (SES)							
HH SES indicator				Measure of impact	Baseline	Follow up	Effect
Monthly total expenditure				% point difference	$420,778	$476,741	13% [31]
Monthly total household expenditure				% point difference	S/.108	S/.144	33% [32]
Monthly total household income				% point difference	S/.90	S/.129	43% [32]
Level 2: Impact on household SES on nutrition							
HH SES indicator	Malnutrition indicator			Measure of impact	Baseline	Follow up	Effect
Monthly household income	Rise in BMI associated with household income between 2 and 5 minimum wages (US$ 290 to US$ 1450) relative to household income less than 2 minimum wages (US$ 290)			Unit increase	–	–	0.8 [33]
Monthly equivalent household income	Rise in BMI associated with a monthly household income increase of MEX$ 1000			Unit increase	–	–	0.12 [34]
Level 3: Impact of nutrition on TB							
Malnutrition indicator	TB indicator			Measure of impact	Baseline	Follow up	Effect
	TB disease	TB diagnosis delay	TB treatment success				
Odds of patients with BMI < 18.5 defaulting treatment relative to patients with BMI 18.5 to 24.9			●	Odds ratio	–	–	2.08 [35]
Odds of patients weighing < 60 kg experiencing > 30 days patient delay		●		Odds ratio	–	–	3.45 [36]
Hazard ratio for TB mortality of BMI < 18 kg/m2 at treatment beginning			●	Hazard ratio	–	–	4.89 [37]
Decrease in TB incidence per unit increase in BMI (BMI range of 18.5 to 30 kg/m2)	●			% point difference			14% [38]

**Table 4:** Challenges encountered during S-PROTECT and research roadmap

Challenge	Specific example	Way forward
*Methodological*	Study population uncertainties in terms of TB patients covered by social protection and extent of mixing between social protection beneficiaries and general population	Additional epidemiological analysis are needed to characterise the target populations of specific CTIs, in relation to the broader populations in which those individuals live. Furthermore, it will be important, in a given setting, to characterise better the poverty profile of TB patients compared to the general population: understand the extent to which they overlap, in which way the differ, how this can influence their likelihood to be seen and targeted by cash transfers schemes, as well as this can predict their response to CTIs.
	No consensus on rules for data harmonisation across different levels and unit of measures	It will be key to identify appropriate ways to standardise the outcomes and units of measure used across impact evaluation and epidemiological studies, so that they can be appropriately utilised in any future research work on the relationship between development and TB. This effort should be linked to the establishment of an open data portal containing reliable and coherent effect estimates for at least some of the identifiable pathways. Considering the vast and diverse literature involved, such an effort is too ambitious for S-PROTECT alone and should become an independent objective of a broader initiative on social protection and TB (and health in general)
*Conceptual*	Only 13 pathways were taken into account: only using a materialistic perspective	Other aetiologic models should be considered. For example, using a psychosocial framework, it could be argued that CTIs may reduce levels of stress in a population or even improve their mental health, which in turn may improve individuals’ immunological function and thus risk of TB infection and reactivation, as well as the way children respond to BCG vaccine. From a life-course perspective, it is indeed possible that at least much of the preventive effect of CTIs on TB may not be measureable in adults benefiting from CTIs today, but on adults that have benefitted from CTIs while they were children. It is increasingly acknowledged that the most damaging effects of poverty on health happen during childhood and today CTIs are mainly targeted at children to break the inter-generational transmission of poverty and to reduce this damaging effect of poverty of the physical and mental development of children [19]. It can be argued that this can also apply to TB in terms of risk of TB infection, risk of progression to TB as an adult, and also risk of childhood TB [39]. Future work could implement a life course model [28] to represent CTIs effects on factors such as malnutrition and children’s immune system development [40] and changes in TB exposure over a lifetime [41], while capturing dynamic population age-shifts in incidence [42]. To understand further the plausibility of these alternative pathways, their measurability and how they can be best incorporated into TB modelling efforts, it will be important to expand further the interdisciplinary nature of S-PROTECT, and engage with experts from other disciplines possibly as part of social protection research networks operating under the same framework and scope, such as the recently established Social Protection Action Research and Knowledge Sharing (SPARKS) network.
	Only 13 pathways were taken into account: issues of generalisability	S-PROTECT efforts need to replicated and adapted to settings other than Brazil to account for both a context-specific social protection environment and different TB epidemic profile.

### The model development

We conceptualised our modelling approach to capture the impact of CTIs – and BFP in particular - on TB in two parts. In the first part, we aimed to estimate the impact of CTIs like BFP on each of the proximal mediators of TB outcomes. This was achieved by first estimating the effects at each of the three levels of a given pathway separately based on the literature review, and then combining them together. In the second part, we aimed to estimate the impact of change in any of the proximal mediators of TB outcomes on the overall TB prevalence. To achieve this, we formulated a simple model of TB transmission and natural history, which factored in each of the proximal mediators of TB outcomes to generate estimates of TB prevalence.

The complete mathematical description of the model, along with the parameters used to calibrate the model and a schematic representation of the model are presented in Additional file 2 (see online Additional file 1: Appendix 3 to see how pathway # is expected to affect the model parameters estimates).

## Results

Based on the secondary data drawn from the literature and our assumptions we estimated that the impact of BFP on the proximal mediators of TB outcomes as mediated via pathway #10 alone to be generally modest. For example, we estimated that BFP would improve TB treatment success by 1.5% (0.28, 4.77%), time to TB diagnosis by 0.12% (0.04, 0.59%), and TB transmission rates (measured as TB incidence) by 1.33% (0.48, 2.89%). The overall modest impact reflected both relatively low increase in household income achieved via CTIs like BFP (15% increase) and low effect of income on nutrition (0.129 increase in BMI per US$1000 increase in household income). The large range in estimates generally reflected uncertainty in data, such as the estimates of BFP driven changes in household income, and the impact of improved nutrition on the proximal mediators of TB outcomes.

We then estimated the change in TB prevalence induced by the changes in each of the proximal mediators of TB outcomes by the quantities estimated above. The change in TB prevalence was calculated with the transmission model at its respective equilibrium, before and after the change, hence providing an estimate for a long-term change. For example, for reduction achieved via pathway #10, we estimated an overall long-term reduction in TB prevalence by 3.9% (low: 0.7%, high: 23.5%). Of this, 1.9% (0.1, 14.0%) reduction was achieved via improved TB treatment success alone, 0.7% (0.1, 6.8%) by reduction in time to treatment alone and, 1.3% (0.5, 2.9%) achieved via reduction in disease susceptibility alone.

We also sought to estimate the direct impact of BFP without restricting the impact to be mediated via effect on income. Using an estimated impact of BFP-equivalent in Mexico on nutrition (which estimated a 0.83 units increase in BMI [19], we then proceeded to estimate the impact on each of the proximal mediators of TB outcomes, and used the same model to estimate the impact on TB prevalence. We estimated an impact of 32.1% reduction in TB prevalence via this direct measure, almost 10 times larger than our estimates from pathways driven by income only (Additional file 2).

## Discussion

The S-PROTECT project has contributed to the increasing bulk of evidence on the impact of CTIs on TB epidemiology and control. Most importantly, it allowed the identification of a number of both conceptual and methodological challenges that must be addressed in future mathematical modelling if we are to successfully develop estimates of the impact of CTIs - and social protection in general - on TB elimination efforts.

These challenges are outlined below and summarised in Table 4. For each of them we also propose a research road map.

### Study population

It is critical to specify the population under study. Most CTIs apply very stringent criteria to target their beneficiaries, based on poverty-related criteria both at household and community level. This target population is often people living in extreme poverty at highest risk of TB disease, but individuals who qualify for CTIs intermingle with people who do not (“general population”) in ways that are very challenging to understand and quantify. Furthermore, the relative size of the target population, as well as the relative prevalence of TB in this population (both compared to the general population) is often poorly defined and mainly available from a limited number of prevalence surveys that have collected socioeconomic data [20, 21]. It is impossible to quantitatively estimate the impact of a given CTIs intervention on TB epidemiology in a general population without specifying (through data or assumption) three quantities: (a) size of the target population (as a proportion of the total population); (b) relative TB burden in the target population (versus the general population); and (c) degree mixing dynamics between members of the target and general population. With S-PROTECT we tried to circumvent these concerns by focusing a modelling effort on the CTI target population alone (i.e. the people eligible for BFP) and effectively assuming no interplay with the general population. With this assumption we challenged the conventional idea of TB incidence “hotspots” defined geographically and replaced it with the notion of hotspots defined by high risk groups bearing the highest burden of disease in a given community. This assumption was deemed legitimate for Brazil, where the epidemic of TB is highly concentrated in some high-risk groups [22], but this approach may be less appropriate in other settings. Nonetheless, it is plausible to think that by intervening in the highest-risk populations (who may generate the majority of TB transmission), ending TB also in the general population may be achieved effectively [23, 24].

### Data availability and harmonisation

Despite the abundant evidence on the impact of CTIs and the vast literature on TB, it was challenging to identify and bring together the necessary evidence for each level of the studied high priority pathways. This was partially due to the fact that we aimed to link data extrapolated from different types of sources and also different levels of analysis and partially because there is currently no consensus regarding how to convert outcomes and units of measure even when they are available. For example, studies that estimate associations between CTIs and structural determinants of health (“level 1”) often use different outcomes than those that study associations between structural determinants of health and proximal mediators of TB (“level 2”) or between those proximal mediators and TB outcomes (“level 3”). Specifically, for Pathway #10, we found that “level 1” studies estimated the impact of CTIs on household SES as a percent change in monthly income or expenditure whilst many “level 2” studies estimated the association between SES and malnutrition, with SES measured as a dichotomous variable (i.e., above or below an income threshold). In this case, to estimate the effect of CTIs on malnutrition via income it would have been necessary to further assume the relationship between a percent increase in income/expenditure crossing the “level 2” studies’ income thresholds. Similarly, whilst most “level 2” studies used a continuous increase in BMI as the dependent outcome, most “level 3” studies used WHO BMI weight categories (< 18.5, 18.5 to 24.9 and > 25) as independent determinants of TB outcomes, thereby requiring two conversion steps to estimate the quantitative impact of social protection on TB outcomes, and a third conversion if the “level 3” outcome did not precisely correspond to a specific parameter in the model. This conversion is even more challenging when the “linking” outcome measures themselves (i.e., monthly expenditure and BMI in the example of Pathway #10 are incongruous.

### Understanding pathways

S-PROTECT aimed to contribute to the understanding of the mechanism through which CTIs act on TB prevention and clinical outcomes; however, as demonstrated this pathway approach may lead to an underestimate of the ultimate impact if the effect is somehow diluted across levels and/or the biggest impact happens at the bottom of the tail. Furthermore, it is unlikely that 13 pathways identified in S-PROTECT were able to capture the full effect. CTIs are likely to improve characteristics of the population in ways that are difficult to analytically define, and these improvements may have important effects on TB dynamics. It is also very likely that the prioritisation exercise we attempted for Brazil may return a different ranking of pathways in a different study setting. Furthermore, study generalisability may be only limited to countries within the Latin American region where social protection programs similar to BFP operate. Nonetheless, generalisability of findings was not a key goal in this stage of the SPROTECT project, whose main scope was indeed the development and testing of a generic mathematical modelling approach that could be adopted and adapted for other settings despite the context-specific pathways ranking and impact findings. Furthermore, TB inequalities have been typically explained using a material/neo-material lens [25], by which TB happens mainly because of the exposure to inadequate material living conditions (i.e. poor housing quality, insufficient or bad quality food consumption, dangerous working conditions, etc) [16]. This was the approach taken by S-PROTECT too; however it is indeed plausible that alternative aetiological models such as the psychosocial [26] or life course approach [27, 28] could also help to explain the marked TB gradient across socioeconomic strata and consequently provide further explanation of TB inequalities are established, perpetrated and can be mitigated. This is also clearly expressed in the theory of fundamental causes of health inequalities proposed by Link and Phelan [29], according to which multiple pathways need to be tested and blocked to achieve a detectable impact at population level.

## Conclusions

To our knowledge the S-PROTECT project is the first attempt to use mathematical modelling to predict the impact social protection interventions, such as the BFP, on indicators of TB prevention, care and control.

The modest estimates of impact on TB prevalence are just illustrative, but reflect generally the low impact of BFP on the proximal mediators of TB, and the consequences of restricting ourselves to estimating the impact via narrowly defined pathways. Furthermore, we may have underestimated the true effect by not accounting for heterogeneity of impact across different groups (i.e. extremely poor household), by assuming an homogenous distributions of TB patients as well as a linear relationship between BFP vs income and income vs nutrition and nutrition vs TB indicators. While these are important limitations worth to be addressed in future analysis, our primary purpose for this paper was to develop a generalizable framework, not to develop exact or comprehensive estimates of BFP impact on TB in Brazil.

The approach we have adopted was helpful to improve our quantitative understanding of the linkages between social protection and TB burden, highlight key gaps in both data and aetiological pathway thinking, and prioritise efforts for data collection and transformation. These emerged lessons will inform the next phase of S-PROTECT, through which we aim to refine our methodological approaches (including impact heterogeneity), expand our focus beyond CTIs (i.e. employment protection and/or housing and slum upgrading interventions) and to develop a more sophisticate, yet still amenable for mathematical modelling, conceptual framework encompassing a life-course approach and accounting also for psychosocial factors. However, for the time being S-PROTECT proved that by combining different expertise, it is indeed possible to model the impact of social protection on TB prevention, care and control with existing knowledge and analytical tools.

## Additional files

Additional file 1: Appendix 1. Data review strategy. Appendix 2. Assumptions for pathway #10. Appendix 3. Model parameters affected by Pathway #10. (PDF 461 kb)

Additional file 2: A) Model development and B) Illustrative example of Bolsa Familia Programme (BFP) impact estimates for pathway #10. (DOCX 112 kb)

## Abbreviations

BFP: Bolsa Familia Programme
BMI: Body Mass Index
CTI: Cash Transfer Intervention
S-PROTECT: Social Protection to Enhance the Control of Tuberculosis
TB: Tuberculosis
WHO: World Health Organization
