# Supplementary material for: Modelling the impact of social protection on tuberculosis: the S-PROTECT project
Source: BMC Public Health. 2018 Jun 26;18:786. doi: 10.1186/s12889-018-5539-x (PMC6020219; doi:10.1186/s12889-018-5539-x)

Appendix 1. Data review strategy

Search Strategy: Separate searches were conducted for each of the levels of each of the three chosen high priority pathways. In each of the searches the search terms were designed to return literature relating to the impact/association of a factor on/with a factor one level closer to the TB outcomes for the priority pathway that the search was related to. For example, for pathway #11 we first searched the literature on the impact of CTIs on household Socioeconomic Status (SES), then the impact of household SES on malnutrition and finally the association of malnutrition with TB reactivation and risk of TB treatment success. The search was restricted to studies in Brazil or Latin American and Caribbean countries, as defined by the World Bank and published after 2000. A summary of the search terms used for each of the reviews is provided in Appendix: Box 1. Whenever possible search results were supplemented with grey literature.

Electronic searches: Information sources for Level 1 and 2 searches included EconLitand Global Health. Information sources for Level 3 searches included Global Health and Pubmed.

Types of intervention: interventions had to be operated by a federal government and consist of direct monetary transfers to households made-up at least partially of a component conditioned on a particular behaviour (e.g. school attendance or visit to health facility for regular check-ups).

Study outcomes:

Level 1:

1. SES measured either as household income or household consumption expenditure.
2. Food security measured either as a self-assessed scale, total calories consumed or food consumption expenditure as a % of total expenditure.
3. Access to health services measured as daily visits to public health facilities.

Level 2:

1. Malnutrition measured by BMI.

Level 3:

1. TB treatment success measured as TB treatment completion or TB cure.
2. TB reactivation measured as reactivation of latent TB infection.
3. TB diagnostic delay measured as patient time in days since onset of symptoms to diagnosis.

Selected studies: One author (WR) independently selected the studies to be included in the review by scanning titles of search results. No restrictions were placed on the types of study design included in the review. The relevance of selected studies was ranked in order of their setting, with Brazil ranked highest followed by the rest of Latin America.

Data extraction and management: For each of the three levels of the pathways effect estimates were extracted into separate standardised extraction tables: one for the impact of CTIs on distal determinants of TB, one for the impact of distal determinants of TB on social determinants of TB and one for the impact of social determinants of TB on TB outcomes.

Appendix 2: Assumptions for pathway #10

Parameter	Best estimate	Low estimate	High estimate	Direct estimate	Comments
Current (or as recent as possible) TB prevalence/100,000 in the population receiving BFP	91	91	91		The estimates are based on TB prevalence for Brazil, adjusted for higher likelihood of TB among people with lower income. We have allowed for lower range to go below the national average of Brazil based on some estimates that find TB prevalence to be lower that national average among Bolsa Familia Participants.[1] WHO estimate of prevalence in Brazil per 100,00 is 52.[2] The multiplier for lowest quintile (compared to middle) is: 1.92 (South Africa[3]); 2.01 (India[4]); 1.32 (Vietnam[5]).
TB treatment success rate (%)	82	72	92		We have used Torrens et al 2016 for the best estimate, and taken the range to include WHO estimate for Brazil (72%).[2]
Median annual household income among people receiving BFP (US $)	4980	3132	7128		116.3 (BR$, per capita, per month): This sample is restricted to families with per capita income less than R$ 210.00.[6] 216.1(BR$, per capita, per month):[7] The point estimate is the average of the two estimate scaled by HH size of 5 and currency exchange rate of 1US$=0.5 B R$.
Number of household members	5	4.5	5.5		
Mean or median BMI index (Kg/m^2) of people receiving BFP	25	21	27		We used a round middle figure from the two estimates. Estimates were derived as follows: Median of 25.9 (Standard Deviation = 4.5):[8] Data is from beneficiaries of PROGRESA in Mexico (A similar CTI to BFP in Brazil)<br>Median 24.11 (IQR: 21.72-26.98).[8]This is calculated from microdata for the national survey of household budgets that can be downloaded from the internet (n=190,159 individuals).
Relative increase (% change) in household income between households receiving BFP vs not	15	10	20		Personal communication from Sergei Soares, Institute for Applied Economy, Brasilia, Brazil
Absolute change in BMI comparing people receiving BFP vs not				0.83	This figure was only available from PROGRESA in Mexico.[8]

Relative change in TB incidence (% change) per unit increase in BMI	13.8	13.4	14.2		Decrease in TB incidence is interpreted as decrease in susceptibility equivalent to 14% decrease in TB incidence.[9] Range correspond to 95% CI reported in the ref.[9]
Change in BMI units per US $ increase in annual household income	0.0001289	0.000114943	0.000142857		Average estimate from[10] and,[11] with range corresponding to the range of the two estimates.Here we have interpreted data from Ward et al, 2015 as .8 unit of increase in BMI for increase in household income from $290 to average of 290 and 1450.[10]

References

1. Torrens AW, Rasella D, Boccia D, et al. Effectiveness of a conditional cash transfer programme on TB cure rate: a retrospective cohort study in Brazil. *Trans R Soc Trop Med Hyg* 2016;110(3):199-206.
2. WHO. Global Tuberculosis Report 2015. Secondary Global Tuberculosis Report 2015 2015. http://apps.who.int/iris/bitstream/10665/191102/1/9789241565059_eng.pdf?ua=1.
3. Ataguba JE, Akazili J, McIntyre D. Socioeconomic-related health inequality in South Africa: evidence from General Household Surveys. *Int J Equity Health* 2011;10:48.
4. Oxlade O, Murray M. Tuberculosis and poverty: why are the poor at greater risk in India? *PLoS One* 2012;7(11):e47533
5. Hoa NB, Wei C, Sokun C, et al. Characteristics of tuberculosis patients at intake in Cambodia, two provinces in China, and Viet Nam. *BMC Public Health* 2011;11:367.
6. Martins AP, Monteiro CA. Impact of the Bolsa Familia program on food availability of low-income Brazilian families: a quasi experimental study. *BMC Public Health* 2016;16(1):827.
7. Cruz M, Ziegelhofer Z. Beyond the income effect: impacts of conditional cash transfer programs on private investments in human capital. Report No.: WPS6867: The World Bank, Washington, United States of America, May 2014.
8. Fernald LC, Gertler PJ, Neufeld LM. Role of cash in conditional cash transfer programmes for child health, growth, and development: an analysis of Mexico's Oportunidades. *Lancet* 2008;371(9615):828-37.
9. Lonnroth K, Williams BG, Cegielski P, et al. A consistent log-linear relationship between tuberculosis incidence and body mass index. *Int J Epidemiol* 2010;39(1):149-55.
10. Ward J, Friche AA, Caiaffa WT, et al. Association of socioeconomic factors with body mass index, obesity, physical activity, and dietary factors in Belo Horizonte, Minas Gerais State, Brazil: The BH Health Study. *Cad Saude Publica* 2015;31 Suppl 1:182-94.
11. Colchero MA, Sosa-Rubi SG. Heterogeneity of income and lifestyle determinants of body weight among adult women in Mexico, 2006. *Soc Sci Med* 2012;75(1):120-8.

Appendix 3. Model parameters affected by Pathway #10.

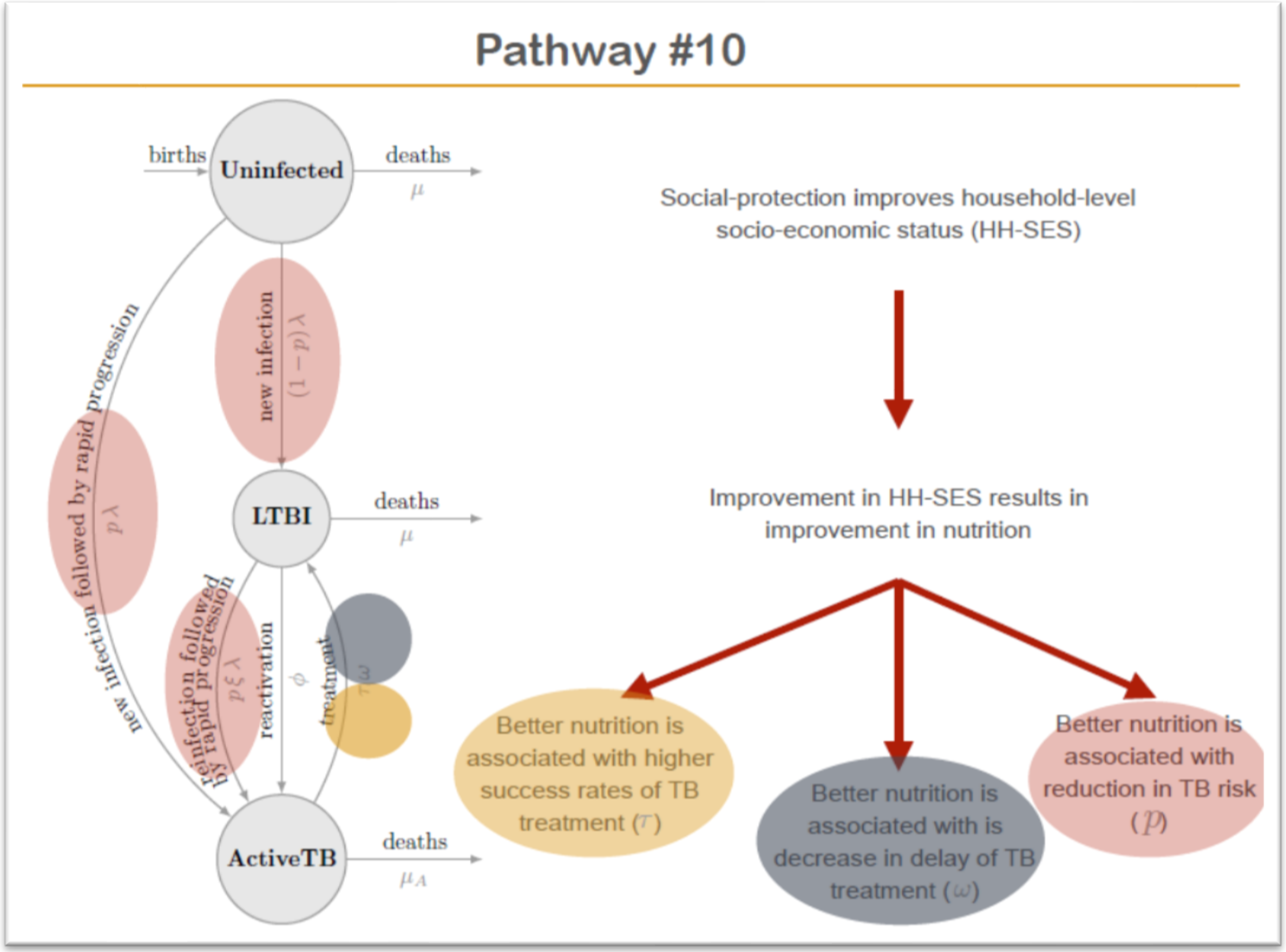

Supplement: Supplementary file 1 — Appendix 1. Data review strategy. Appendix 2. Assumptions for pathway #10. Appendix 3. Model parameters affected by Pathway #10. (PDF 461 kb) [file 12889_2018_5539_MOESM1_ESM.pdf]
