# Supplementary material for: Modelling the impact of social protection on tuberculosis: the S-PROTECT project
Source: BMC Public Health. 2018 Jun 26;18:786. doi: 10.1186/s12889-018-5539-x (PMC6020219; doi:10.1186/s12889-018-5539-x)
Supplement: Supplementary file 2 — A) Model development and B) Illustrative example of Bolsa Familia Programme (BFP) impact estimates for pathway #10. (DOCX 112 kb) [file 12889_2018_5539_MOESM2_ESM.docx]

**Additional file 2. A) Model development and B) Illustrative example of Bolsa Familia Programme (BFP) impact estimates for pathway #10**

Differential equations that describe TB transmission are as follows:

$$\frac{dU}{dt}=\mu L+ \mu_{A} A- \lambda U$$

$$\frac{dL}{dt}=\left( 1-p \right)\lambda U-\left( \phi+ \mu+p\xi\lambda\right)L+ \omega\tau A$$

$$\frac{dA}{dt}=p\lambda\left[ U+\xi L \right]+\phi L-\left[ \omega\tau+\mu_{A} \right]A$$

The three compartments are: $U:$ Uninfected; $L:$ LTBI; and $A:$ Active TB disease.

1. **A simple compartmental model of TB transmission**

In a typical TB transmission model, individuals can be in one of the three compartments with respect to their TB status: Uninfected (U), Latently Infected with TB (L), and carrying active TB disease (A). Individuals are born uninfected, and when exposed to TB either develops LTBI, or active TB disease via rapid progression of the infection. Individuals with LTBI can develop active TB disease either via endogenous reactivation of the latent infection or via rapid progression of a reinfection. Individuals with active TB disease upon receiving proper diagnosis and successful treatment are considered recovered from TB disease; recovered individuals are assumed to have similar risk of TB as individuals currently infected with LTBI.

The parameters that describe the dynamics are: $\mu:$ background mortality rate, 0.02 per year; $\mu_{A}:$ mortality rate among individuals with TB, 0.12 per year; births taken to maintain a stable population; $\lambda:$force of TB infection = (transmission rate) * $A$, where the transmission rate is calibrated to a baseline TB prevalence of 91/100,000; $p:$ probability of rapid progression, 12%; $\phi:$ reactivation rate, 0.001 per year; $1-\xi:$ immunity imparted by previous infection, 60%; $1/\omega:$time to treatment, 1 year; $\tau:$ treatment success probability, 82%.

1. **Estimates of impact: an example from pathway #10**

We estimated the impact of BFP on TB prevalence, by first estimating effects at each of the three levels of a given pathway (and pathway #10 specifically in this instance). We then combined the effect across all levels to estimate the impact of BFP on each of the proximal mediators of TB outcomes.

| **Levels of impact** | | **Best estimate** |  | **Low estimate** |  | **High estimate** |
| --- | --- | --- | --- | --- | --- | --- |
| Level 1 | The impact of CTI^1^ intervention like BFP on household income^2^ | +15% |  | +10% |  | +20% |
| Level 2 | The impact of income on nutrition^3^ | 0.13 |  | 0.11 |  | 0.14 |
| Level 3 | The effect of nutrition on TB outcomes: |  |  |  |  |  |
|  | TB treatment^5^ | 15.6% |  | 7.8% |  | 23.4% |
|  | TB diagnosis^6^ | 1.3% |  | 1.2% |  | 2.9% |
|  | TB progression^7^ | 13.8% |  | 13.4% |  | 14.2% |

| **Combined effect of BFP on individual level TB outcomes^8^** | **Best estimate** |  | **Low estimate** |  | **High estimate** |
| --- | --- | --- | --- | --- | --- |
| % Decrease in treatment failure proportions | 1.50 |  | 0.28 |  | 4.77 |
| % Decrease time to diagnosis and treatment | 0.12 |  | 0.04 |  | 0.59 |
| % Decrease in TB incidence per unit increase of BMI | 1.33 |  | 0.44 |  | 2.89 |

1. CTI: Cash Transfer Intervention
2. Percentage difference in household income comparing beneficiary with non-beneficiary households.
3. Change in Body Mass Index (BMI) per US$1,000 increase in household income
4. Change in Body Mass Index (BMI)comparing beneficiary with non-beneficiary households.
5. % Decrease in treatment failure rate per unit increase in BMI
6. % Decrease in time to TB diagnosis per unit increase in BMI
7. % Decrease in TB incidence per unit increase in BMI taken as reduction in rate of rapid progression to TB
8. The combined effect for each TB outcomes is the product level-specific

Finally, by incorporating the Level 3 estimates into the TB transmission model, we estimated the impact of BFP on long-term TB prevalence.

| **Combined effect of BFP on population level TB prevalence** | **Best estimate** |  | **Low estimate** |  | **High estimate** | |
| --- | --- | --- | --- | --- | --- | --- |
| % Reduction on population-level TB prevalence | 3.9% |  | 0.7% |  | 23.5% |  |
